# TSHZ1-dependent gene regulation is essential for olfactory bulb development and olfaction

**DOI:** 10.1172/JCI163087

**Published:** 2022-08-15

**Authors:** Daniela Ragancokova, Elena Rocca, Anne M.M. Oonk, Herbert Schulz, Elvira Rohde, Jan Bednarsch, Ilse Feenstra, Ronald J.E. Pennings, Hagen Wende, Alistair N. Garratt

Original citation: *J Clin Invest*. 2014;124(3):1214–1227. https://doi.org/10.1172/JCI72466

Citation for this corrigendum: *J Clin Invest*. 2022;132(16):e163087. https://doi.org/10.1172/JCI163087

The description of the primers for ChIP and the size of the Prokr2 PCR fragment were incorrect in the Methods. The correct text is below.

We showed results for 2 sets of primers: promoter (5′-CACACTCCAACTCTGCACACTTAGT-3′ and 5′-ACTGAATTAGGGTCTCAGACTTCCG-3′; 154 bp), which served as a negative control for binding in the promoter sequence, and intron 1 (5′-TTCAGGGCCATTTCAAGCACGGCTA-3′ and 5′- ATACCCTCTTCTGGCCTCAGACAGT-3′; 235 bp), which served to detect a putative binding site of TSHZ1 in the first intron of *Prokr2*.

In addition, we have corrected errors in the text in the legend of Supplemental Figure 1C and in the Supplemental Methods in the description of the generation of mice.

The authors regret the errors.

## Version 1 08/15/2022

Electronic publication

